# 477. Increased Referrals for New PFAPA (Aphthous Stomatitis, Pharyngitis, Adenitis) Diagnosis During the COVID-19 Pandemic

**DOI:** 10.1093/ofid/ofab466.676

**Published:** 2021-12-04

**Authors:** Bessey Geevarghese, Shan Sun, Ravi Jhaveri

**Affiliations:** 1 Ann & Robert H. Lurie Children’s Hospital of Chicago, Elmhurst, Illinois; 2 Northwestern University Feinberg School of Medicine; Ann & Robert H. Lurie Children’s Hospital of Chicago, Chicago, Illinois

## Abstract

**Background:**

COVID-19 pandemic caused by SARS-CoV-2 resulted in a global health crisis in 2020. Quarantining, wearing masks and physical distancing- key infection prevention strategies implemented to stop the spread of COVID-19, also led to dramatic decreases in rates of common respiratory viral infection seen in young children. Due to lack of school and daycare exposure, we evaluated a larger than usual number of patients with periodic fevers without any known infectious contacts. Based on this observation, we conducted an analysis of all suspected cases of periodic fevers seen at our institution during the COVID-19 lockdown compared to prior seasons.

**Methods:**

The clinical charts were queried for all patients presenting to any Lurie Children’s Hospital outpatient specialty clinic or laboratory with ICD diagnosis code of MO4.1 and MO4.8 (all recurrent and periodic fever syndromes) from June 1, 2020 through September 30, 2020, and compared to similar months the previous 2 years (2018 and 2019). Each patient chart was reviewed by the lead investigator to verify all new diagnoses of PFAPA. The number of new patients with PFAPA diagnosis were tallied and analyzed. Statistical comparisons were made using Kruskal-Wallis tests for monthly distributions in different years.

**Results:**

We noted a significant increase in patients with new PFAPA diagnosis between June through August 2020 compared to similar months in 2018 and 2019 (Figure1). Experienced pediatric infectious disease physicians and rheumatologists diagnosed majority of the cases. During these months, a monthly median (IQR) of 13 (11.5, 14.5) patients were diagnosed among different Lurie specialty clinics, which is more than 2.5 folds increase in new PFAPA patients from the previous two years which were about 5 (3.5, 6) (Figure 2).

Number of Patients with New PFAPA Diagnosis

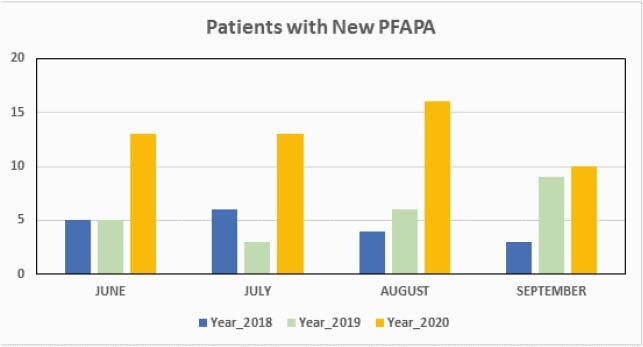

There was a significant increase in number of new patients diagnosed with PFAPA between June through August 2020 compared to similar months in 2018 and 2019.

Monthly Distribution Summary for New PFAPA Diagnosis

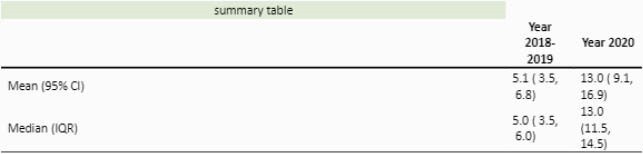

Statistical comparisons were made using Kruskal- Wallis tests for monthly distributions in different years

**Conclusion:**

We observed a significant increase in PFAPA patients referred to our institution soon after introduction of public health measures to slow spread of COVID-19. Given that most children were not in daycare, schools, or camps, we suspect that parents and pediatricians were able to recognize patterns of periodic fevers in children much quicker than preceding years, when fevers would typically be attributed to an infectious process.

**Disclosures:**

**Ravi Jhaveri, MD**, **AstraZeneca** (Consultant)**Dynavax** (Consultant)**Elsevier** (Other Financial or Material Support, Editorial Stipend as Co-editor in Chief, Clinical Therapeutics)**Seqirus** (Consultant)

